# Meeting the challenges to adolescent well-being: context, returns on investment, and financing

**DOI:** 10.1093/heapol/czag083

**Published:** 2026-09-30

**Authors:** Peter Sheehan, Bruce Rasmussen, David A Ross, Anshu Mohan, Susan Sawyer, Bhavya Nandini, Levi Singh, Kim Sweeny, John Symons, Rose September

**Affiliations:** Victoria Institute of Strategic Economic Studies, Victoria University, PO Box 14428, Melbourne, Victoria 8001, Australia; Victoria Institute of Strategic Economic Studies, Victoria University, PO Box 14428, Melbourne, Victoria 8001, Australia; Stellenbosch University, Institute for Life Course Health Research, Im Kloster 28, 76332 Bad Herrenalb, Germany; World Health Organization South-East Asia Regional Office, Family and Gender Health, World Health House, Indraprastha Estate, Mahatma Gandhi Marg, New Delhi 110 002, India; Centre for Adolescent Health, Royal Children’s Hospital, 50 Flemington Rd, Parkville, Victoria 3052, Australia; Geoff and Helen Handbury Chair of Adolescent Health, University of Melbourne, Parkville, Victoria 3052, Australia; Partnership for Maternal, Newborn and Child Health, 20, Avenue Appia, Geneva 1211, Switzerland; The RHR Africa Trust, 225 Jan Smuts Avenue, Parktown North, Johannesburg 2196, South Africa; Victoria Institute of Strategic Economic Studies, Victoria University, PO Box 14428, Melbourne, Victoria 8001, Australia; Victoria Institute of Strategic Economic Studies, Victoria University, PO Box 14428, Melbourne, Victoria 8001, Australia; Accelerated Programme of Action for Children, The Presidency of the Republic of South Africa, Union Buildings, Government Street Arcadia, Pretoria 0001, South Africa

**Keywords:** health and education, human capital, adolescent well-being, economic modelling, low- and middle-income countries (LMICs), benefit-cost ratio (BCR)

## Abstract

Adolescents in many countries face serious challenges in realizing the level of well-being that is their human right. Many factors—deep-seated inequality within and between countries, rapid change in skill requirements for employment, climate change, and social media—are driving these deepening challenges. This paper develops three points about the need for an urgent response to this situation. First, there is now evidence that a powerful suite of cost-effective investments is available to improve adolescent well-being. To support this claim, we draw on recent modelling of investments for many low- and middle-income countries (LMICs), as well as on detailed case studies of three countries (India, South Africa, and Colombia). In all these studies, most of the investments are highly cost-effective, with benefit-cost ratios over 6, and in many cases much higher. However, the cost of these investments is substantial, especially for those that involve the education system, raising major financing issues for most countries. Second, the case studies highlight, at least for South Africa and Colombia, the extent to which within-country inequality harms adolescent development. For example, if school quality is poor for low-income groups or regions, schooling can perpetuate rather than arrest inequality, which in turn can lead to disruptive violence for these groups or regions. Third, countries with limited fiscal space, high debt levels, and inequality and violence curtailing growth rates will find it hard to fund the investments needed to transform adolescent well-being. Major reforms to development finance are necessary, such as those outlined at the Fourth International Conference on Financing for Development at Seville in mid-2025, as well as changes to the priority given to domestic investments. Steps to reduce income inequality by new fiscal measures are also important, to fund the investments in adolescent well-being and to make them more effective.

Key messages

- In spite of valuable progress in some areas, the outlook for adolescent well-being in many countries is now bleak, unless there are major new investment programs. This reflects many factors, including climate change and the low level of learning being achieved in these countries.
- There is now strong evidence of high economic and social returns to packages of investment to promote adolescent well-being, but these come at a high cost.
- High inequality within many such countries both adversely affects adolescent well-being and makes it more difficult to improve their well-being.
- Countries face serious challenges in financing the investments required, especially in the light of the partial withdrawal of the USA from development aid. Current efforts to renew the global financing for development framework are promising, and low- and middle-income countries should be closely involved with them. Countries also need to explore innovative ways to expand domestic financing capacity, including by obtaining more revenue from higher income groups, and to give higher priority to adolescents in planning investments.

## Introduction

This paper starts by reviewing the difficult situation facing in low- and middle- income countries (LMICs). We show that the outlook is now generally bleak, in terms of adolescent well-being broadly defined. In terms of responses to this outlook, we highlight three points. First, recent work has shown that highly cost-effective packages of interventions are available to improve adolescent well-being, but that these interventions are costly in the context of the many other challenges facing these countries. Secondly, there is clear evidence from two countries (South Africa and Colombia) that the returns to schooling interventions are significantly higher in lower than in higher income schools. This implies that giving priority to disadvantaged schools may, as well as being justified on normative grounds, increase overall national returns to these initiatives. Thirdly, the final section of the paper discusses issues concerning the financing of the interventions, in terms of development finance, debt, and the reorientation of national priorities.

### Situation analysis of adolescent well-being in low- and middle-income countries

Over the past two decades, there have been improvements in various aspects of adolescent well-being in many LMICs. For example, the proportion of persons aged 10–19 years enrolled in upper secondary school in LMICs has risen from 50.8% in 2004 to 63.8% in 2023 (UIS 2024). The number of child marriages (marriage before the age of 18 years) has fallen in many LMICs. In terms of global data, the number of child marriages has fallen from nearly 12 million 20 years ago to just over 10 million, with the proportion falling from 23% of girls to 19% (UNICEF 2023). The rate of injuries and deaths from road traffic accidents for 10- to 19-year-olds has also declined strongly, with deaths falling by 30% since 2004 (Global Burden of Disease Collaborative Network 2024). In 2023, 418 million children and adolescents in 176 countries received school meals worldwide, 30 million more than the 388 million reached before the COVID-19 pandemic in early 2020 (WFP 2022). These meals, together with the supplements included with them, help to provide a stronger nutritional base for learning and growth.

Finally, adolescents have been gradually receiving better health care and improved access to health services, leading among other things to a fall in the burden of communicable diseases. Table 1 shows the change in DALYs (disability-adjusted life years) lost among adolescents in all LMICs between 2000 and 2020, by different types of disease (Global Burden of Disease Collaborative Network 2024). For both communicable and related diseases and injuries, the DALY rate for adolescents in this large group of countries fell by around 40% between 2000 and 2020. While not down to levels in high-income countries (HICs), this is a major improvement. The DALY rate for adolescents in LMICs for non-communicable diseases fell by 6.1% over the period. While this fall is small relative to the 40% drop seen across the other groups, it compares favourably with a rise, also of 6.1%, for HICs over that time.

**Table 1:** DALY rate for persons aged 10–19 years, LMICs, 2000 and 2020.

	2000	2020	% change, 2000–20
Communicable, maternal, neonatal, and nutritional diseases	6678	3911	−41.4%
Non-communicable diseases	6459	6062	−6.1%
Injuries	3571	2270	−36.4%

LMICs, low- and middle-income countries.

Source: Estimates of the authors based on Global Burden of Disease Collaborative Network (2024).

In spite of these and other gains reflecting the efforts of communities, governments, and agencies, by the middle of the third decade of the twenty-first century the outlook for adolescent well-being in many LMICs is bleak, unless there is substantial new investment. This disturbing outlook arises from the impact of large-scale global changes affecting adolescents, together with failures in some critical programs serving young people. While many factors are relevant, here we briefly note four which have a significant effect: climate change and mental health, learning poverty, technological change and changes in employment, and persistent inequality.

#### Climate change and mental health

It is now clear that continuing climate change, manifesting both in the growing frequency of dangerous weather events and in projections of future warming, is adversely affecting adolescent mental health in many countries, as well as the physical conditions for young people in some regions. As the world continues to warm, these effects are likely to become more marked and of deepening concern to younger generations. This is not to deny that many other factors are also shaping the crisis in mental health, perhaps especially in developed economies.

Adolescents are at a stage of life that involves significant biological, psychological, and social changes, making them particularly vulnerable to the impacts of a changing climate on both physical and mental well-being (McGushin et al. 2022, Meherali et al. 2025). The impact of climate change on the physical health of adolescents includes increased risks of heat-related illnesses, injuries, respiratory conditions including asthma, allergies, and waterborne diseases due to changes in precipitation patterns. Some adolescents also suffer from an increased burden of anxiety, depression, trauma, and post-traumatic stress disorder due to the uncertainty and stress linked with climate change impacts (van Nieuwenhuizen et al. 2021). These impacts include displacement and forced migration from natural disasters or apprehension about future catastrophes (Meherali et al. 2025).

A recent analysis of national data sources in the USA and UK has shown an abrupt decline in the mental health of the young, and in their subjective well-being, in those countries since about 2010 (Blanchflower et al. 2025). For the USA, the key measure is the incidence of ‘despair’, which occurs when the survey respondents answer that their mental health was not good for all 30 days prior to the interview. For persons aged 18–24 years, the rate of despair rose steadily in the USA from 3.21% of respondents for females and 2.50% for males in 1993, to 8.24% for females and 5.09% for males in the final pre-COVID year 2019, increasing further to 9.32%/6.63% by 2023/4, respectively. Similar trends were observed for the UK. Analysis by Blanchflower et al. (2025) of data from 44 countries from the Global Minds Project (Sapien Labs 2025) for 2020–25, which includes the COVID period, shows that the incidence of mental distress is higher for persons aged 18–24 years, than for all other age-groups in 43 of the 44 countries.

#### Learning poverty

While rates of attendance at school have risen in almost all countries, there is now evidence that the learning achieved by many adolescent populations has plateaued and is far from acceptable in many countries. The focus on the expansion of school attendance has often been at the expense of the quality of learning in schools and the development of skills and other aspects of human capital (Pritchett 2024). There are two main empirical approaches to documenting the extent of the learning crisis, both based on analysis of the many surveys now undertaken on school attendance and performance across countries.

First, the World Bank and the United Nations Educational, Scientific and Cultural Organization (UNESCO) have assembled data on a simple literacy competency test, namely, whether a child finishing primary school can read and understand a simple sentence in their own language. Learning poverty is defined as the proportion of children of the relevant age who are either not in school or who fail the reading test. They found that in 2019, prior to the COVID-19 pandemic, 57% of children in LMICs suffered from learning poverty. The pandemic had an adverse effect on schooling and learning, and the two institutions estimate that by 2022 the rate of learning poverty in LMICs had increased to 70% (World Bank 2022).

Gust et al. (2024) have undertaken a similar exercise, but covering skills in mathematics and science as well as reading, and for students in secondary schools at about 15 years of age. They worked with a large number of data sets and anchored their measures to mastering at least the most basic level of the Programme for International Student Assessment (PISA), i.e. PISA Level 1 skills (OECD 2020). They used imputation and other methods to obtain estimates for countries without survey data and for children not in secondary school. They found that about two-thirds (67.2%) of the world’s children at this stage do not have basic PISA 1 skills, and that for LMICs the proportion is close to 90%. There is indeed a sharp divide in these estimates between countries at different income levels: for low-income countries (LICs) the share without PISA 1 skills is 95.6% and for LMICs it is 85.8%, whereas for upper-middle-income countries it is 42.3% and for HICs it is 25.5%.

#### Technological change and changes in employment

In a world in which both economic structures and technologies are changing rapidly, such low levels of literacy, numeracy, and related skills provide a poor base from which to address the challenges facing today’s adolescents. For example, manual and routine jobs are being replaced by new technologies across the board; the service and care sectors are displacing the goods sector, especially as sources of employment, while emerging artificial intelligence technologies imply a new era of change. Employment will remain a major challenge for young people, especially for those without a quality education and the development of strong skills that are necessary to succeed in the labour market.

#### Persistent inequality

Pronounced inequality, both within and across countries, remains a persistent, and in some respects, an increasing reality in the world (Chancel et al. 2022). For about 160 years (from 1820–1980), inequality across countries steadily increased, as what are now the HICs captured the fruits of the industrial revolution within a framework of colonization. But this trend changed markedly around 1980, as a number of large countries, such as China and India, started to grow rapidly. This period of more rapid growth, with opening up to global markets and private development, has led to a sharply increasing within-country inequality in many countries, as illustrated for South Africa and India in Fig. 1.

**Figure 1:**
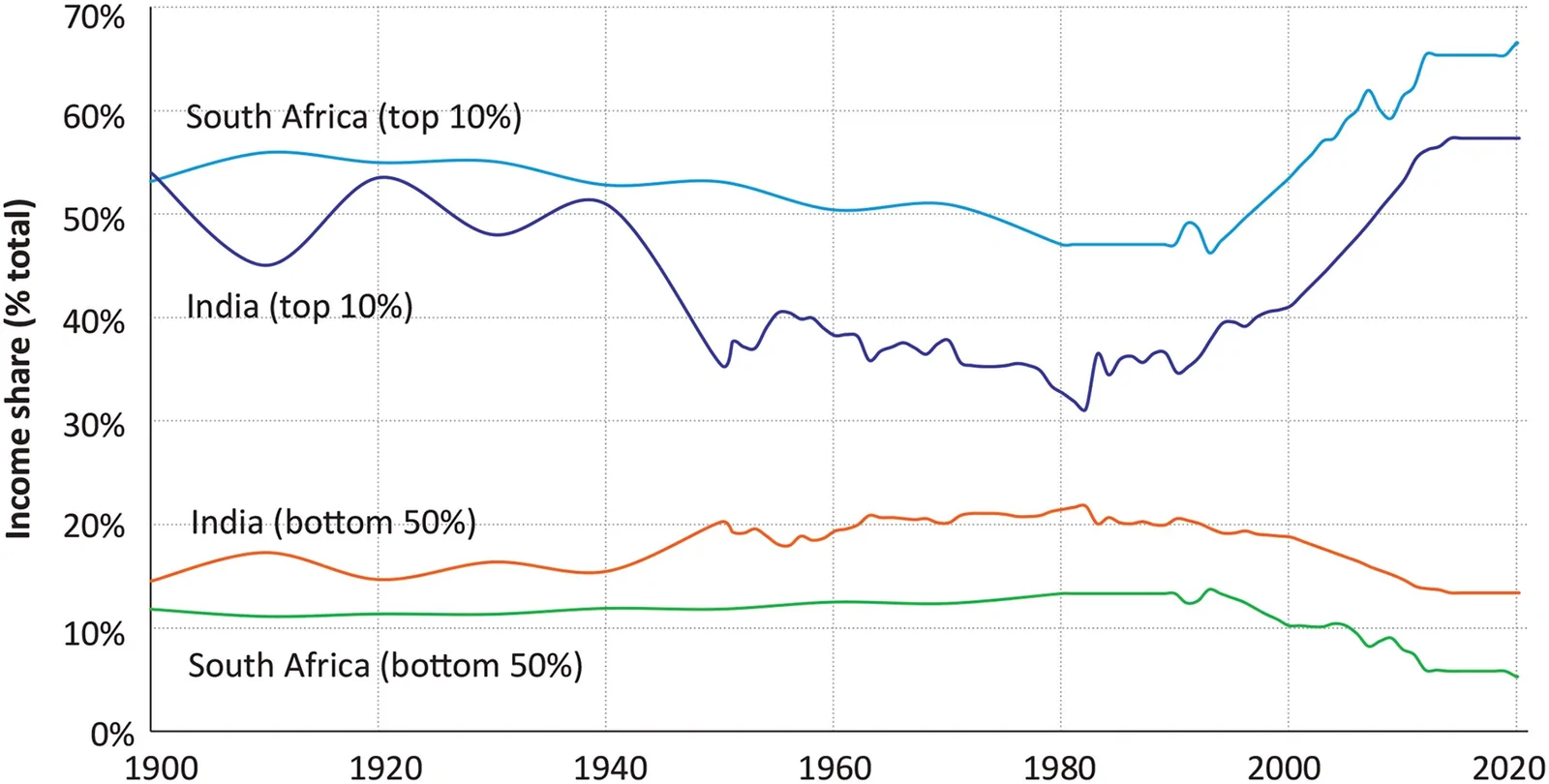
Shares of total income for top 10% and bottom 50% of the population, South Africa and India, 1900 to 2020. Source: Authors’ estimates based on Chancel et al. (2022).

Adolescent well-being is strongly related to socioeconomic status, whether within countries or between them (Dierckens et al. 2020, Elgar et al. 2024). As is to be expected, adolescent well-being is, on average, lower in LICs than in HICs and, within a country, lower in the lower socioeconomic groups than in higher socioeconomic groups.

## Materials and methods

### Modelling methods

Given developments in both science and policy, a powerful suite of investments is available to improve adolescent well-being in ways that are strongly cost-effective. We provide evidence for this conclusion by reporting on the latest results from a decade of modelling of such investments for a wide range of LMICs, undertaken by the authors and their colleagues. These latest results are drawn from four reports prepared by the current authors and other colleagues for the Partnership for Maternal, Newborn and Child Health (PMNCH) (VISES 2024, 2025a, 2025b, WHO 2024). Two studies by other parties are also reported.

#### Multi-country study

For this study, seven models were developed or redeveloped for specific programs of intervention to improve adolescent well-being. These have been used to measure the cost, impact, and benefits resulting from these investments, for countries and interventions for which there are sufficient data available on benefits and costs to allow calculation of benefit-cost ratios (BCRs). We focus on BCRs (the ratio of the estimated value of benefits achieved to costs incurred) as the form of assessment used in the studies being reported here. Estimation of the monetary valuation of the benefits achieved is necessary to calculate a BCR.

Table 2 provides a summary of the development history of these seven models and two others included, namely: 5. Verguet et al. (2020) and 6. Stelmach et al. (2022). In many cases, these models build upon and extend earlier models developed in conjunction with colleagues, especially from the United Nations Population Fund (UNFPA), World Health Organization (WHO) (including PMNCH), and the United Nations Children’s Fund (UNICEF) in previous studies. We wish to acknowledge the contributions made by the various co-authors of earlier papers and the role of the agencies, especially UNFPA and PMNCH, which commissioned and, in part, funded these earlier studies.

**Table 2:** Origins and development paths for the nine benefit-cost models for which results are reported in this study.

Model origin and development	Development work for this report
**Health interventions to 2035**	
This model studies 90 adolescent interventions for 40 LIC and LMIC countries. It consists of an epidemiological model based on the UN OneHealth Tool plus an economic model to estimate BCRs. It was built for Sheehan et al. (2017) and reported in Sweeny et al. (2019).	Full re-estimation of OneHealth Tool (OHT) results with updated cost and economic components. See WHO (2024, section 2.1).
**Specialist health programs—single interventions**	
2. HPV. The model used here addresses the impact of vaccination to prevent and ultimately eliminate cervical cancer in women. It has both epidemiological and economic components.	The current model was built jointly with the Daffodil Centre, University of Sydney, and Sweeny, Nguyen et al. (2023). See WHO (2024, section 2.2).
3. Tuberculosis (TB). This model estimates the BCR from implementing the WHO End TB Strategy in 50 countries where 95.7% of adolescent TB deaths occur.	For further details of this analysis see WHO (2024, section 2.3).
4. Myopia screening and treatment. This model estimates the return to interventions to address cataracts and refractive error in 19 countries, mainly in Asia and Africa, where incidence is high.	Developed with the support of the Fred Hollows Foundation, the model uses inter alia evidence from the Lancet Global Health Commission on Global Eye Health (Burton et al. 2021). See WHO (2024, section 2.4).
**Specialist health programs—multiple interventions**	
5. Nutrition (school meals). Verguet et al. (2020) developed a model to estimate the cost-effectiveness of school meals, with supplements to treat worms and anaemia in 14 developing countries.	The model estimates both the costs and impact of school meals and calculates the benefits from improved health, learning, and food production.
6. Wide-ranging mental health interventions. Stelmach et al. (2022) developed a Markov model to follow 10- to 19-year-old cohorts in 36 countries being treated for anxiety, depression, bipolar disorder, and suicide risk.	This analysis covers a mix of both high-income countries and LMICs, giving substantial attention to treatments delivered online.
**Interventions to 2050**	
7. Education and training. Built for Sheehan et al. (2017) from earlier work by UNESCO and UNICEF; reported in Wils et al. (2019). The schooling model traces the student transition through grades, subject to interventions at various points. An economic model estimates the value of better schooling and skills.	The results reported here come from an updated version of the Wils et al. (2019) model, with a new meta-analysis for effect sizes and the grouping of related interventions. See WHO (2024, section 6.2).
8. Prevention of child marriage. Initial modelling reported in Sheehan et al. (2017) and Rasmussen et al. (2019) with further developments in Rasmussen et al. (2021).	The model used here now includes 70 countries with updated data inputs and effect size estimates. See WHO (2024, section 7.2.2).
9. Road traffic injury prevention. Initially developed for Sheehan et al. (2017), see also Symons et al. (2019). Further enhanced since then with support from the FIA Foundation (Symons and Sweeny 2021).	For WHO (2024), the model was substantially upgraded from the 2022 version, with a new approach to the baseline and to rural/urban analysis, updating of effect sizes, and better infrastructure modelling. See WHO (2024, section 7.4).

There are two other points worth noting about these models. First, they adopt, in several different forms, a broadly human capital approach to valuing the benefits. A central aspect is the potential gross domestic product (GDP) lost from death, illness, or injury with, where relevant, the additional social value lost through death, illness, or injury, and/or the potential GDP gained from improved human capital. These estimates are prepared using country- and age-specific projections of participation rates and death rates (WHO 2024).

Secondly, for multi-country studies, two methods are available for reporting the average ratio of benefits to costs, i.e. the average BCR. The first is simply to add up the BCRs for each country and divide by the number of countries. This provides the ‘unweighted average’, in which the estimated BCR of each country has the same weight. The second is to sum up all the estimated benefits separately and then compile the total costs across countries and divide the total benefits by the total costs. This ‘weighted average’ BCR gives a much bigger weighting to countries generating larger estimated total benefits and total costs (e.g. those with larger populations such as China and India). Both of these averages are reported in Table 3, where they are available. In addition, we also report in these tables the results of modelling for mental health for 36 countries (Stelmach et al. 2022), and for nutrition and school meals for 14 countries (Verguet et al. 2020), which have been reported by other research groups.

**Table 3:** Return on investment for adolescents, benefit-cost ratios from multi-country studies.

Intervention programmes	Weighted BCR	Unweighted BCR	Number of countries covered
**Health interventions to 2035**			
1. Adolescent health services (90 programs)	5.4	9.8	40
**Specialist programs—single interventions**			
2. Human papillomavirus (HPV) vaccination	17.8	20.2	78
3. Tuberculosis (TB) treatment	54.4	53.6	50
4. Myopia screening and treatment	21.1	13.0	19
**Specialist programs—multiple interventions**			
5. Nutrition (school meals with supplements) (Verguet et al. 2020)	17.0	Not available	14
6. Wide-ranging mental health interventions (Stelmach et al. 2022)	23.6	Not available	36
**Interventions to 2050**			
7. Education and training	15.9	28.6	64
8. Child marriage prevention	25.9	30.3	70
9. Road injury prevention	9.1	13.4	77

The right-hand column shows the number of countries modelled for each intervention programme. The choice of countries is determined by the availability of data and other modelling considerations.

Sources: Health and other areas (WHO 2024). Mental health (Stelmach et al. 2022). Nutrition (Verguet et al. 2020).

Thirdly, there are a number of other relevant features of these models which should be clarified:

- the perspective of the BCR is in all cases the societal perspective;
- all costs and benefits are included, as far is possible, as specified more fully in WHO (2024);
- there is no specific target audience, as the BCRs should be available to all interested parties;
- the time horizons are as specified in Table 2 and in the text; and
- the discount rate used is 3%.

While there is no agreed benchmarking for BCR values in health studies, in many contexts a BCR of 3 or more (total benefits 3 or more times total costs) is regarded as a strong signal for investment.

Two key issues are important in interpreting the BCR results reported here. In all cases, a base case (an interpretation of unchanged policy) is established, and then a series of interventions is applied to that base case, with the benefits of the interventions calculated as the value of the difference in outcomes between the two cases. The two issues that differ between the models are the period for which the interventions are applied and the specification of the base case. For technical reasons, such as model or data availability or differences in the estimated lag times for the impact of the interventions to manifest themselves, the approach to these two issues varies across the models. In all cases, the benefits are calculated across a longer period than the costs, normally the projected working life of the individuals involved, to capture the increase in lifetime earnings associated with the intervention.

For the four health models (Models 1–4 in Table 2), the interventions are applied up to 2035, being progressively scaled up relative to the base case over the period. For the education, child marriage, and road traffic injury models (Models 7, 8, and 9, respectively, in Table 2), we apply the interventions up to 2050.

#### Three national case studies: Colombia, India, and South Africa

The models used in these multi-country studies have been applied, with refinements, to three national case studies: Colombia, India, and South Africa. As with the multi-country study, these analyses confirm high BCRs for many investments, but also highlight three critical issues on which further work is ongoing—inequality, violence, and mental health.

The case study countries span three continents, Africa, America, and Asia. India is by far the largest of the three with a population in 2024 of 1.45 billion and a gross national income (GNI) of US$3840 billion (World Bank 2025a). While the World Bank (2025b) estimates that the population of India will continue to grow to 1.62 billion by 2040, due to falling birth rates over the past two decades, the number of adolescents is projected to decline from close to its peak of 252 million in 2024 to 222 million by 2040.

Despite their different histories, South Africa and Colombia have surprising similarities. Their economies, with a GNI of US$390 billion and US$373 billion and populations, 64 million and 53 million in 2024, respectively, are not dissimilar (World Bank 2025a). In other ways, however, they are currently moving in different directions with South Africa’s population growing younger, while Colombia’s is ageing (UN 2024a). The number of adolescents in Colombia is projected to decline from 7.2 million in 2024 to 7.0 million in 2040, while South Africa’s is expected to continue to increase from 10.7 million in 2024 to 11.8 million by 2040 (UN 2024a).

As shown in Fig. 2, the health status of adolescents, as measured by the DALY rate (disability-adjusted life years lost per 100 000 population) for all causes, has been generally improving globally, and substantially so for the three case study countries (Global Burden of Disease Collaborative Network 2024). The DALY rate for Indian adolescents has fallen by 35% from 17 653 DALYs in 2000 to 11 475 in 2021, comparable to the global average. The DALY rate also fell in the other two countries, Colombia (by 29%) and South Africa (by 12%).

**Figure 2:**
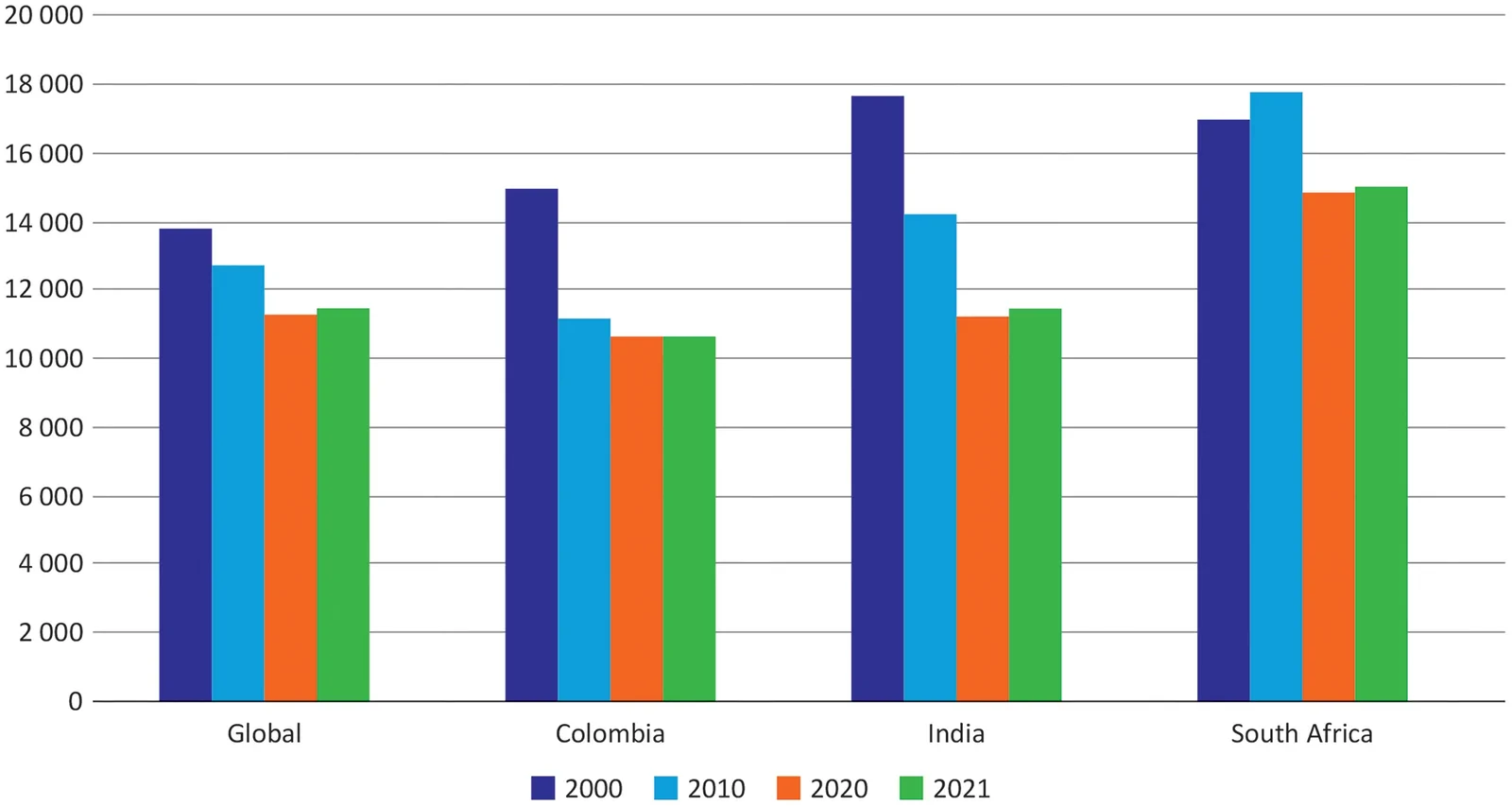
Health status of adolescents globally and for case study countries, DALY rate, all causes 2000, 2010, 2020, and 2021. Source: Based on data from Global Burden of Disease Collaborative Network (2024).

Adolescents in both South Africa and Colombia suffer from particularly high burdens of interpersonal violence and above global average levels of mental disorders. It is likely that the two are related. Measured by burden of disease, adolescents in both countries suffer from about the same level of mental disorder, a DALY rate of 1843 for Colombia and 1882 for South Africa (Global Burden of Disease Collaborative Network 2024). While the DALY rate for interpersonal violence has declined substantially in both countries, it remains the highest disease burden for adolescents in both countries.

## Results

### Summary of modelling results

#### Multi-country study

Table 3 provides a summary of the results from the multi-country study for packages of investments to improve adolescent well-being. Only summary results can be provided here, with much greater detail provided in the original reports. This table uses the same numbering system as Table 2, so that the results can be compared with the characteristics of the specific model used.

It is notable that all of the reported BCRs are above 5 on a weighted average basis and above 9 on an unweighted basis. These are very high returns for any social investment analysis, indicating that the benefits are estimated to exceed the costs many times over. The estimated benefits are benefits to society as a whole, and are not realized by the agency funding the interventions.

They are also expensive investments. The average annual cost to 2035 for the investments described in Table 3, excluding those on mental health and nutrition conducted by other research groups, is US$222 billion. While precise calculations are difficult because of the diversity of programs and countries, this amounts to about 0.52% per annum of the GDP of the relevant countries.

It should be noted that there are many differences in the nine modelling exercises reported in Table 3, although broadly similar modelling approaches are used. Some of these differences arise from the nature of the interventions themselves, ranging from low-cost interventions such as for tuberculosis (TB) to complex, costly interventions for schooling. Others arise from different modelling assumptions, and the interaction of a common 3% discount rate with interventions with varying time profiles. What is striking about the results in Table 3 is that all of the BCRs are relatively high, with most over 15. Care should be taken in comparing BCRs across interventions, as a more detailed study of each estimate is required to draw valid comparative conclusions.

#### Three case studies

As with the multi-country estimates, our objective was to estimate the returns to investing in adolescents across as many of the main dimensions of adolescent well-being as we could, using the available intervention parameters. While this work focused on adolescent health and education, it also included reducing violence (bullying), child marriage, and road traffic injuries.

The estimated BCRs are shown in Table 4. For health, they show the differences in BCRs between the multiple intervention programmes such as other adolescent health services (which includes 95 specific programs, as detailed in VISES (2024)), and specialist programs such as TB treatment and screening, and treatment for myopia. The specific specialist programs modelled have relatively low cost and large impact, and therefore high BCRs. For example, for TB treatment in India and South Africa, where the prevalence is relatively high, the BCRs are well above the global average at 71.4 and 99.0, respectively.

**Table 4:** Benefit-cost ratios, multi-country, and case study countries.

Interventions	Multi-country	India	Colombia	South Africa
**Health interventions to 2035**				
Adolescent health services (SRH, etc.)	5.4	5.9	4.9	8.5
**Specialist programs—single interventions**				
2. Human papillomavirus (HPV) vaccination	17.8	16.7	Not available	Not available
3. Tuberculosis (TB) treatment	54.4	71.4	37.9	99.0
4. Myopia screening and treatment	21.1	15.5	26.1	31.4
**Specialist programs—multiple interventions**				
5. Mental health (treatment of depression and/or anxiety)	23.6^a^	Not available	15.3	10.5
**Interventions to 2050**				
6. Education and training	15.9	14.4	9.5	18.1
7. Child marriage	25.9	19.9	18.6	6.6
8. Road injury prevention	9.1	4.6	4.3	7.3
Violence (bullying)	12.3	16.2	14.3	17.3

^a^ Stelmach et al. (2022) include bipolar disorder and suicide, whereas the VISES estimates for Colombia and South Africa are limited to the treatment of depression and anxiety.

Source: Authors’ estimates.

Similarly for education and training in India and South Africa, where the secondary school completion ratio is low, the BCRs are high at 14.1 and 18.1, respectively, reflecting the large benefits obtained from lifting the quality and years of education attained in these two countries. The BCR for child marriage prevention programs is high for both India and Colombia, 19.9 and 18.6, respectively, but nonetheless lower than the multi-country average of 25.9. Child marriage in both countries is already trending down, and therefore the effect of the additional interventions is muted by this. Reported child marriage is low in South Africa, hence the low BCR. But teenage pregnancy is a major issue in South Africa and can be addressed by similar programs.

Adolescent interpersonal violence is common in the three case study countries, although less so for India. Both South Africa and Colombia fall within the top 10% of countries based on GBD estimates of the burden from interpersonal violence (Global Burden of Disease Collaborative Network 2024). The available evidence for modelling BCRs for interpersonal violence is limited, but modelling the costs and benefits of adolescent bullying, a major indicator of violence, has been undertaken. As shown in Table 4, the BCRs were uniformly high and for the three case study countries, were above the global average.

### The impact of inequality on the returns to education

The results in Table 4 conceal the large differences in rates of return for different income groups. Income inequality for South Africa and Colombia is among the highest in the world. The estimated Gini coefficient for South Africa (63.0) and for Colombia (53.9) are the highest and third highest, respectively, of any country recorded by the World Bank (2024).

The returns to education are generally higher for schools serving low-income groups, compared with high-income groups, where the gap in service quality is large. Table 5 shows the return on investments related to education (BCRs) for the lower three and upper two income quintiles in South Africa and the rural/urban split (proxy for lower-/upper-income groups) for secondary schools in Colombia. It shows that the return on investment in the schools primarily serving lower-income groups is estimated to be approximately twice that of investing in schools serving the upper-income groups. As already indicated, the return on investment in South Africa is higher than in Colombia, largely due to the higher intervention costs and an already higher secondary completion rate in Colombia.

**Table 5:** Returns on investment in education, costs, benefits, and benefit-cost ratios, 2024–50.

	South Africa			Colombia		
	Lower 3 quintiles	Upper 2 quintiles	Total	Rural	Urban	Total
NPV costs (US$ billion)	13.8	21.2	35.0	7.0	46.0	53.0
NPV benefits (US$ billion)	352.4	280.9	633.3	121.3	383.8	505.1
BCR	25.5	13.2	18.1	17.4	8.3	9.5
BCR (sensitivity case +/− 5%)	24.3	14.1	18.1	16.6	8.6	9.7

BCR is the benefit-cost ratio. NPV is the net present value which is the discounted sum of future costs and benefits. A discount rate of 3% is used.

Source: Authors’ estimates.

For South Africa, the BCR for lower-quintile schools is estimated to be 25.5 compared with 13.3 for the upper-quintile schools. For Colombia, the rural/urban split is estimated to yield BCRs of 17.3 and 8.3, respectively. These estimates indicate that there are very high returns to adolescents disadvantaged by income and/or location. But they are based on an assumption that the employment income earned by students completing a given education level (e.g. year 12) does not vary across income or location of schooling. This assumption may not be correct. That is, students completing year 12 in a high-income area may have better earnings prospects than those completing year 12 in a low-income area. To test the sensitivity of our results to this effect, we report in Table 5 the results of a sensitivity analysis in which earnings for a given level of school completion are 10% lower for the low-income group than for the high-income group. The results, shown in the last line of Table 5, are that the finding of higher returns in low-income groups remains strong, albeit somewhat reduced in the sensitivity case.

Education has long been regarded as one of the strongest instruments for reducing poverty (World Bank 2025c). However, the evidence from these case studies suggests that the current provision of education in Colombia and South Africa reflects the existing inequalities and acts to entrench them (VISES 2025a, 2025b).

In South Africa, the schooling outcomes and teaching experience of adolescents from the upper two income quintiles, who largely attend fee-paying schools, are substantially different from those of adolescents from the lowest three income quintiles, who largely attend state schools serving low-income families (NDoH 2019). For example, in 2016, secondary school completions among the upper two income quintiles were 66% compared with those from the lowest three quintiles, which were only 34% (NDoH 2019).

For Colombia, the education outcomes for urban and rural schools, which also largely represent high-income and low-income groups, are very different. On average, the years of schooling for adolescents living in urban areas in 2016 are 10, and for those living in rural areas are only 8. Secondary school completions for urban areas were 76% compared with only 44% in rural areas (DHS 2016).

The school infrastructure, teaching resources, and quality of the pedagogy are often lower in the schools attended by students from low-income quintiles or rural families. From a low base, the interventions to improve both the access to and quality of education provided to adolescents in the rural (Colombia) or low-quintile (South Africa) populations are estimated to have a marked effect on reducing dropout rates and improving school completions, generating large benefits compared with relatively modest costs. This issue of differential returns to interventions across the income spectrum is an important one for further research.

## Discussion

It is necessary to recognize the limitations of these estimates. For example, given the many assumptions that must be made in the calculations, there is a considerable band of uncertainty around the results, for which we cannot provide a statistical assessment. This is a genuine limitation of this analysis and further work is necessary, both by ourselves and others in the field, to develop better measures of the uncertainty bound around a given BCR estimate. The linkages between the different interventions need further study, as attacking different areas of disadvantage in a coordinated way may have different, and perhaps stronger, effects than addressing each individually.

Nevertheless, the results do indicate that powerful and effective investments are available to countries with the funding, expertise, and political will to implement them. But they are expensive investments, especially for countries with limited fiscal space, low economic growth, and high debt levels. As noted above, the estimated average annual cost of the investments to increase adolescent well-being that were included in the multi-country study is US$222 billion, or about 0.52% of GDP per annum on average. Costs may be higher or lower, in both dollar terms or as a share of GDP, for different countries, and their impact on the well-being of poorer adolescents may be reduced by the effects of systemic inequality and the violence to which it gives rise.

Financing the necessary investments in adolescent well-being is a critical issue for many countries. Limited fiscal space, high debt levels, and the impact of inequality and violence on growth rates are among the factors that make such financing difficult. This issue is a component of the broader challenge facing the international community in financing the investment needed to fund the Sustainable Development Goals (SDGs). The funding gap to achieve the SDG goals has recently been estimated at US$4 trillion annually (UN 2024b).

To address this funding gap, the UN and other parties set up the Fourth International Conference on Financing for Development (ICFD4), held in Seville, Spain, on 30 June–3 July 2025. Among other things, the Conference reported as follows:

We decided to launch an ambitious package of reforms and actions to close this financing gap with urgency and catalyse sustainable development investments at scale. We will take concrete actions to enhance fiscal space, address debt challenges of developing countries and lower the cost of capital. We will provide and mobilize additional, innovative, adequate, affordable, predictable and accessible financing from all sources, recognizing the comparative advantages of public and private finance. (UN 2025, p2, paragraph 6)

To implement this and other related decisions, the assembled governments and agencies decided to pursue action in seven areas:

- domestic public resources, including effective taxation of natural resources;
- domestic and international private business and finance, including the development of innovative financial instruments that are scalable and involve blended finance;
- international development cooperation and development effectiveness, including South-South cooperation;
- international trade as an engine of development, including the defence of the multilateral trading system;
- debt and debt sustainability;
- international financial architecture and system issues, including allocation of Special Drawing Rights (SDRs) by the IMF and rechannelling of SDRs from developed to developing countries; and
- science, technology, innovation, and capacity.

This is a complex but promising agenda, which defines many of the key steps that need to be taken. But the world has a sorry history of important reports that have gathered dust rather than being implemented. The policies of the second Trump administration in the USA towards development finance and trade must be regarded as a setback to this agenda, although there are signs of greater commitment from other countries. While much remains to be done on the design and evaluation of initiatives to improve adolescent well-being, the issue of how to finance these initiatives needs to be brought to the fore.

Governments and agencies need to be closely involved in the follow-up to ICFD4, working with other countries and international agencies on programs that might assist in financing critical social investments, such as those discussed here in relation to adolescent well-being.

Equally, a focus on developing domestic fiscal space to address the urgent needs of adolescents is vital. This should involve increased taxation on higher income groups, given the pervasive nature of serious inequality in so many countries. A redirection of fiscal priorities from less productive areas to meet the needs of young people is also necessary. The second Lancet Commission on Adolescent Health and Wellbeing (Baird et al. 2025) analysed, for a group of countries, the proportion of global development assistance for health that went to adolescents (here defined as those aged 10–24 years). They found that the share was only 2.4%, while adolescents in those countries were one quarter of the population and carried 10% of the disease burden.

An important additional finding from the two national investment cases where this was explored (Colombia and South Africa) was that the estimated return on investment (BCR) for interventions to improve the access to, and quality of, education was roughly double for adolescents in the lower three wealth quintiles than in the upper two quintiles, and in the adolescents living in rural versus urban areas in South Africa and Colombia, respectively (Table 5). This would argue that countries should prioritize equitable domestic and foreign investment in education and training, improving access to, and the quality of, free or heavily subsidized education and training for institutions serving the poorest adolescents. This policy decision should be based not only on moral principles and human rights arguments (Sen 1999, UNESCO 2016), but also on the fact that it is where the best returns on investment are likely to be found.

## Data Availability

The data underlying this study were obtained from publicly available third-party sources. Details of the datasets and their access links are provided in the relevant references listed in the reference list.

## Reflexivity statement

The research originated from a global study into the urgent need for investment in adolescent well-being including health and education especially in LMICs. The authors include four women and five men with professional experience across multiple levels and countries. All authors specialize in various areas of public health and health policy. This study has benefitted greatly from the considerable expertise of the authors across the globe and their work across varied geographies within public health practise, economic modelling and analysis, research and policy making.
